# Functional patient-derived cellular models for neuropsychiatric drug discovery

**DOI:** 10.1038/s41398-021-01243-8

**Published:** 2021-02-17

**Authors:** Santiago G. Lago, Jakub Tomasik, Sabine Bahn

**Affiliations:** Department of Chemical Engineering and Biotechnology, University of Cambridge, Cambridge, United Kingdom

**Keywords:** Drug discovery, Psychiatric disorders, Pharmacology, Pathogenesis, Biomarkers

## Abstract

Mental health disorders are a leading cause of disability worldwide. Challenges such as disease heterogeneity, incomplete characterization of the targets of existing drugs and a limited understanding of functional interactions of complex genetic risk loci and environmental factors have compromised the identification of novel drug candidates. There is a pressing clinical need for drugs with new mechanisms of action which address the lack of efficacy and debilitating side effects of current medications. Here we discuss a novel strategy for neuropsychiatric drug discovery which aims to address these limitations by identifying disease-related functional responses (‘functional cellular endophenotypes’) in a variety of patient-derived cells, such as induced pluripotent stem cell (iPSC)-derived neurons and organoids or peripheral blood mononuclear cells (PBMCs). Disease-specific alterations in cellular responses can subsequently yield novel drug screening targets and drug candidates. We discuss the potential of this approach in the context of recent advances in patient-derived cellular models, high-content single-cell screening of cellular networks and changes in the diagnostic framework of neuropsychiatric disorders.

## Perspective

### Current bottleneck in neuropsychiatric drug discovery

Major neuropsychiatric disorders represent a substantial burden on worldwide health, accounting for 31% of years lived with disability (YLD)^1^ and a lifetime prevalence of over 20% of the global population (approximately 17% for major depressive disorder, 2.4% for bipolar disorder and 1–2% for schizophrenia and autism depending on geographic region^2–4^). They are associated with significant comorbidities including cardiovascular disease, suicide, substance abuse, immune disorders, obesity and diabetes^1,3^. Current treatments are effective in only 40–60% of individuals^5,6^, providing symptomatic relief as opposed to a cure. Other limitations include debilitating side effects, such as over-sedation and delayed-onset of therapeutic efficacy^3,6^. Despite this urgent medical need, no drugs with fundamentally new mechanisms of action have emerged for over two decades^6,7^ and many pharmaceutical companies have abandoned their neuropsychiatric R&D initiatives altogether^7^.

This is largely because there is a fundamental lack of understanding with regards to the pathophysiology of neuropsychiatric disorders which has compromised the identification of novel drug targets^7^. The major neuropsychiatric medications share mechanisms of action, including effects on monoaminergic neurotransmission^7^, with compounds that were discovered serendipitously in the 1950s and 1960s^6,7^. Since then the pharmaceutical industry has focused on the development of a vast array of monoaminergic drug derivatives with improved efficacy, safety or administration profiles^6–8^. However, because the fundamental mechanisms of drug actions have remained similar, specific patient subgroups and symptom spectra (such as negative symptoms in schizophrenia) which were refractory to first generation drugs have not been addressed by newer generation monoaminergic drugs^3,7^. Likewise, the tenuous relationship between behavioral traits in preclinical animal models and neuropsychiatric symptoms in humans is often validated using existing monoaminergic drugs^6,7^, further precluding any mechanistically novel pharmacophores. Finally, the full mechanisms of action of many of the monoaminergic drugs and non-specific binding to off-target receptors are yet to be characterized^6,7,9^.

**Fig. 1:**
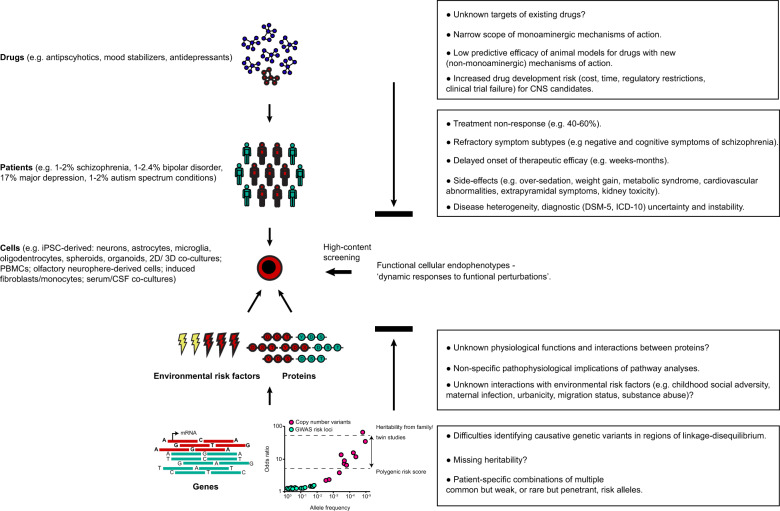
Translation gap in neuropsychiatric drug discovery. The figure summarizes the major obstacles and pending questions in neuropsychiatric drug discovery (boxes right) at the drug, patient, environmental risk factor, protein and gene levels. Disease heterogeneity, diagnostic uncertainty and incomplete characterization of the molecular targets of current neuropsychiatric medications have led to many patients who either do not respond to treatment, present with treatment-refractory symptom domains or suffer from debilitating side effects. On the other hand, the genomic complexity of neuropsychiatric disorders, in terms of multiple common but weak or rare but penetrant risk alleles (shows schematic distribution of allele frequencies vs. odds ratios for GWAS risk loci and copy number variants adapted from ref. ^3^), unknown susceptibility loci (missing heritability) and uncharacterized interactions between genetic and environmental risk factors, in addition to incomplete functional annotation of protein interaction databases, has made it difficult to accurately prioritize potential drug targets at the cellular level based on molecular profiling data. Functional testing of patient and control cells using ligand libraries and high-content screening provides a means to summarize the integrated effects of multiple molecular and environmental risk factors (red) as convergent abnormalities in cellular response (‘functional endophenotypes’) which may represent more physiologically relevant drug targets for specific patient subgroups.

Only recently have primary targets of existing neuropsychiatric drugs, such as the dopamine 2 receptor (*DRD2*) or glutamate receptor subunits (*GRM3*, *GRIN2A*, *GRIA1*) in schizophrenia, been linked to genetic risk of disease at the population level through large-scale **genome-wide association studies** (GWAS; see Glossary)^10^. However, polygenic risk scores explain only a fraction of genetic disease liability, for example 7% in schizophrenia^3^ relative to 64–81% heritability derived from family and twin studies^11^. Moreover, putative individual GWAS risk alleles account only for a marginal increase in disease risk with odds ratios typically under 1.1 and differences in allele frequencies between cases and controls often less than 2%^10,12^. The concept that each neuropsychiatric patient presents with a different combination of multiple common but weak, or in some cases rare but penetrant, risk alleles^3^ has led to the use of in silico pathway analyses to identify cellular pathways which may represent convergent drug targets at the population level^13^. However, this approach is hindered by the fact that expression quantitative trait loci (eQTL), protein function and pathway analysis databases are insufficiently annotated to provide meaningful functional analyses relative to the molecular and cellular complexity of the human brain. Moreover, these resources often implicate non-specific pathophysiological alterations such as cell motility, glycolysis, synaptic plasticity or differentiation^13^, which are too general to represent ‘druggable’ targets. This is compounded by a limited understanding of how complex environmental risk factors, such as childhood social adversity, maternal infection, urbanicity, migration status or substance abuse, interact with genetic risk loci (**gene-environment interactions**) to impact disease etiology, onset and progression^3,14,15^. Thus, despite the wealth of molecular profiling data accrued in recent years it is very hard to translate these insights into functional target-based drug discovery (Fig. 1).

A final limitation is that the patient profiling strategies applied to date lack the dimensionality of a true systems biology approach, in that they do not measure the strength of interactions between molecular risk factors and how they change over time to impact integrated disease phenotypes at the cellular or physiological level. The dynamic nature of disease processes and loss of homeostatic coping mechanisms can only be assessed empirically if individual patient-derived samples are subjected to multiple system perturbations or functional challenges with kinetic resolution^16^.

### **P**atient-derived cellular models of neuropsychiatric disorders

Drug target discovery in neuropsychiatric disorders has historically focused on the pathophysiology of the central nervous system (CNS) using post-mortem brain tissue, neuroimaging or animal model paradigms. While these approaches have added to our understanding of the disorders, they lack the vital feature of being able to assess dynamic cellular changes in relevant human tissue. However, the emerging concept that neuropsychiatric disorders are systemic disorders with corresponding manifestations in the brain and peripheral tissues^17–20^ suggests that different cellular models derived from peripheral cells could offer an unprecedented opportunity to screen for functional drug targets in relevant patient-derived tissue.

**Induced pluripotent stem cells** (iPSCs), created by introducing key pluripotency genes into adult somatic cells, have received considerable attention in recent years as a potential source of patient-derived cellular models for neuropsychiatric disorders, including schizophrenia, bipolar disorder, autism spectrum condition, Timothy syndrome, Fragile X syndrome and major depressive disorder^21,22^. iPSCs have been reprogrammed into a variety of different brain cell lineages, including cortical-excitatory, hippocampal and inhibitory neurons, microglia, oligodendrocytes and astrocytes^21,22^. Importantly they have demonstrated putative disease hallmarks, such as altered neuronal connectivity in schizophrenia or neuronal hyperexcitability in bipolar disorder, which were reversed by antipsychotic and mood-stabilizing medications, respectively, suggesting that they could potentially predict clinical drug efficacy. Recent developments in this field have concentrated on scaling-up iPSC-derived cultures to form more complex multi-dimensional cell networks which enable spatial interactions between different cell-types to be explored. These include co-cultures of microglia-mediated synaptic pruning^23^, microfluidic hippocampal synapses^24^, neural spheroids and brain organoids. Brain organoids have furthermore displayed a diversity of brain cell types, photosensitivity and complex cortical-like features^25,26^ and have been used to study complex developmental processes such as neuronal progenitor proliferation, interneuron migration and cortical layer formation^21^. The use of brain organoids is still in early stages for neuropsychiatric disorders. For example, one single-cell RNA sequencing study reported altered GABAergic specification and Wnt signaling in brain organoids derived from monozygotic twins discordant for schizophrenia^27^. Another study reported downregulation of pathways involved in synaptic biology, neurodevelopment and cell adhesion, concurrent with reduced stimulation and depolarization responses in brain organoids from individuals with bipolar disorder^28^. Nevertheless, organoids have been successfully employed in a number of other disease indications such as drug repurposing screens against Zika virus, SARS-CoV-2 infection modeling and precision medicine for cystic fibrosis and a range of cancers^29^.

While these iPSC-derived models represent an unprecedented opportunity to explore neuropsychiatric cellular alterations in relevant CNS tissue with the genetic background of patients, they continue to face several limitations. These include difficulties in selection of the iPSC colonies, specificity of end fate differentiation, intra-patient variability of iPSC clones, karyotypic instability across passages and differential power requirements for idiopathic versus monogenic gene variants^21,22^. These are compounded in the case of organoids by differences in intrinsic versus directed patterning and the inability to mature to postnatal stages, potentially due to lack of vascularization^21^. Together, these features have meant that this approach remains relatively high-cost, variable, and low-throughput.

Cells which share many of the characteristics of brain cell lineages can also be induced directly from primary patient tissue without the need for reprogramming, including neuronal-like cells from fibroblasts^30^, microglial-like cells from peripheral monocytes^31^ and olfactory neurosphere-derived cells^32^. Finally, CNS cell lines or cells from control donors can be cultured in patient-specific body fluids, for example using patient-derived serum or cerebrospinal fluid, to investigate the effects of disease-associated secreted factors^33^.

It is also possible to use primary peripheral cells in their native state. **Peripheral blood mononuclear cells** (PBMCs) are possibly the best example of this application. They are both accessible for sampling and amenable to high-content screening in suspension^34^. Consequently, they represent a scalable model with the potential to satisfy the power requirements of neuropsychiatric disease investigations whilst facilitating the depth of cellular exploration necessary to reveal complex disease processes in their native state. The majority of investigations using PBMCs in neuropsychiatry have focused on determining the relative proportions of different cell subsets, their activation status or their cytokine secretion profiles^35,36^, consistent with hypotheses of immunological dysfunction in these disorders, and more recently on interactions with the human microbiome^19^. However, recent data suggests that PBMCs can also provide a surrogate model for exploring systemic alterations in a subset of CNS drug targets. Subtypes of CNS receptors (e.g., dopamine and 5HT receptor subtypes) and their cell signaling substrates (e.g., Akt1 and GSK-3β) have been shown to be altered in the brain, as well as PBMC subsets of neuropsychiatric patients and correlated with therapeutic efficacy or disease severity^17,37–39^. GWAS data also suggests the enrichment of single nucleotide polymorphisms associated with neuropsychiatric (schizophrenia) risk loci within PBMC subtype-specific gene expression enhancers^10^. Moreover, PBMCs have shown preliminary evidence of parallel epigenetic changes to those observed in the brain following exposure to environmental stressors, such as early life social adversity^15,40^, raising the possibility of exploring drug-target interactions which are specific to environmental risk factors. Although many of the pathways which are shared between PBMCs and CNS cells are likely to respond differently and the degree of functional overlap between lineages remains to be fully determined, recent evidence suggests that subsets of pathways (e.g., calcium signaling via PLC-γ) or even individual protein-protein interactions, which do overlap, might serve as a proxy for clinically relevant targets which are otherwise inaccessible in primary patient samples^41^. Likewise, it is possible that, at least in a subpopulation of patients, targeting proteins which mitigate immune dysfunction may contribute to symptom remission, as exemplified by the modest efficacy of celecoxib in clinical trials involving first-episode schizophrenia patients with predominantly positive symptoms^42^.

### The functional cellular endophenotype strategy for neuropsychiatric drug discovery

**Fig. 2:**
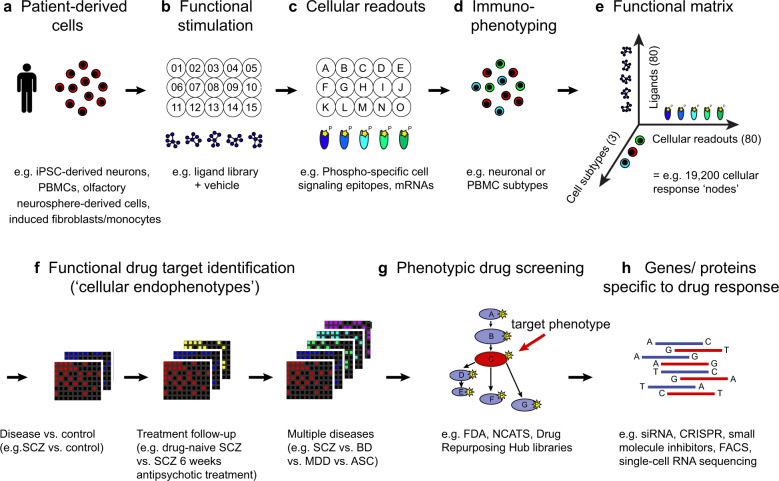
Neuropsychiatric drug discovery pipeline based on functional cellular endophenotypes. **a** Human patient-derived cells (e.g., iPSC-derived neurons, PBMCs, olfactory neurosphere-derived cells and induced fibroblasts/monocytes) provide an accessible ex vivo model of physiologically relevant single-cell phenotypes in health and disease. **b** Patient-derived cells are stimulated using ligand libraries (e.g., 01–15) and vehicle. **c** Single-cell responses to the ligands are measured relative to the vehicle condition across an array of cellular readouts (e.g., phosphorylation of cell signaling epitopes or mRNA expression; A–O) and (**d**) multiple cell subtypes (e.g., neuronal or PBMC subtypes; red, blue and green) using high-content screening (e.g., flow cytometry, mass cytometry, high-throughput microscopy, multiplexed ion beam imaging and single-cell RNA sequencing). **e** This produces a combinatorial expansion of the number of functional assays (e.g., 80 ligands × 80 cellular readouts × 3 cell subtypes = 19,200 assays) performed in each patient-derived cell sample with each ligand-readout-cell subtype combination defined as a cellular response ‘node’. All nodes together provide a profile of the functional repertoire of the cells from each donor. **f** Comparison of the node profiles between donors in different clinical groups allows the identification of abnormal cellular responses (**functional endophenotypes**) which are altered in the disease state (disease vs. control comparison), normalized by efficacious clinical treatment (treatment follow-up comparison) and specific to subsets of neuropsychiatric disorders (multiple disease comparison). **g** These abnormal functional endophenotypes (e.g., depicted in red on pathway A-G) can be targeted through phenotypic drug screening (red arrow) of compound libraries to identify novel drug candidates capable of restoring normal cellular network function. **h** Further mechanistic dissection of genes and proteins underlying the functional interaction between disease endophenotypes and drug mechanisms of action can be conducted using siRNA, CRISPR-Cas9 and small-molecule inhibitor counter-screens, fluorescence-activated cell sorting (FACS) and single-cell RNA sequencing. SCZ schizophrenia, BD bipolar disorder, MDD major depressive disorder, ASC autism spectrum condition, FDA US Food and Drug Administration, NCATS National Center for Advancing Translational Sciences, CRISPR clustered regularly interspaced short palindromic repeats gene editing.

The **functional cellular endophenotype** (see Text box) strategy aims to directly identify abnormal functional responses in patient-derived live cells, relative to healthy individuals, and subsequently use these responses as novel drug screening targets (Fig. 2)^41^. First, live cells (e.g., iPSC-derived neurons, PBMCs; Fig. 2a) from patients and controls are incubated with mechanistically diverse ligand libraries (e.g., CNS receptor agonists, cytokines, hormones, growth factors, antigens or intracellular signaling modulators; Fig. 2b). Second, responses for each ligand treatment relative to the vehicle are assessed across multiple functional readouts (e.g., phosphorylation of cell signaling proteins or mRNA expression) in parallel using single-cell high-content screening (e.g., flow cytometry, mass cytometry, high-content microscopy or single-cell RNA sequencing; Fig. 2c). Third, immunophenotyping is used to resolve responses across different cell subpopulations (e.g., PBMC subsets or iPSC-derived cell subtypes; Fig. 2d) within the heterogeneous cell sample. This creates a combinatorial expansion of the number of functional assays performed in each cell sample (Fig. 2e). Each ligand-readout-cell subtype combination represents a cellular response ‘node’. All nodes together provide a profile of the functional repertoire of the cells from each donor. In addition, the same matrix can be applied at different time points or with different ligand doses to provide kinetic resolution or functional titration of the cellular responses.

Comparison of these node profiles between donors in different clinical groups (Fig. 2f), for example neuropsychiatric patients vs. healthy controls, allows the identification of cellular responses which are altered in the disease state. Crucially, the disease-associated cellular responses can then be targeted through phenotypic drug library screening to derive novel drug candidates capable of normalizing these responses (Fig. 2g). Finally, clinically relevant disease mechanisms linked to drug responses can be elucidated by follow-up genomic or proteomic experiments (Fig. 2h).

The application of this strategy is particularly relevant for tackling complex disorders, such as neuropsychiatric conditions. The use of patient-derived cells provides a unique opportunity to model the genomic and epigenomic complexity of neuropsychiatric disorders in a physiologically relevant context. Recent data suggests that the genetic architecture of neuropsychiatric disorders consists of multiple common but weak or rare but penetrant genetic risk factors, some of which are inherited while others may be sporadic (or ‘de novo’)^3,10,43^. Moreover, each patient likely has a different combination of these risk factors. It is therefore plausible that drug targets are best represented at the pathway level where integrated effects of these diverse risk factors are likely to converge^44^. These distinct downstream abnormalities in pathway responses (functional endophenotypes), which are shared by subgroups of patients despite divergent genetic backgrounds, represent an opportunity to summarize genetic heterogeneity, in addition to environmental risk factors, at a time when functional interactions between risk variants are currently too complicated to model or even unknown. Examples of functional endophenotypes include altered calcium responses in T cells at PLC−γ1 linked to *ATP2A2* polymorphisms in schizophrenia^41^ or spontaneous calcium hyperexcitability in dentate gyrus-like neurons derived from iPSCs in bipolar disorder^45^. Moreover, the use of functional testing in live cells allows the elucidation of relevant disease-specific alterations in cellular networks (or pathways) which are not reflected by quantitative changes in mRNA or protein levels in their basal state, as demonstrated by glycolytic pathway alterations following antigenic stimulation in schizophrenia patient PBMCs^46^. This includes perturbations in homeostatic and regulatory mechanisms consistent with the concept of altered ‘**cellular coping**’^47^.

### High-content single-cell functional screening

High-content screening technologies, such as flow cytometry^48^, mass cytometry^34^, high-throughput microscopy^49^, and single-cell RNA sequencing^50^, enable the depth of functional exploration necessary to identify endophenotypes in neuropsychiatric patient-derived cells^41^. The simultaneous detection of multiple readouts (e.g., signaling protein phosphorylation^34^ or mRNA expression^50^) in individual cells following diverse ligand stimulation allows the readouts to be correlated across thousands of single-cell measurements in each sample. This can serve to generate hypotheses as to causative signaling relationships and alterations in network connectivity associated with disease at the target discovery stage, for example increased negative regulation within the Akt1 pathway in CD4+ T cells from autism spectrum condition and schizophrenia patients^51^. Moreover, changes in the phosphorylation activation status of key therapeutic targets can be normalized relative to total protein abundance or mRNA expression, a feature that has recently revealed novel mechanisms of action for the mood stabilizer lithium in iPSC-derived neurons from patients with bipolar disorder^52^. The ability to measure multiple markers at the single-cell level also affords the statistical power necessary to identify clinically relevant functional phenotypes in minority cell sub-populations within a heterogeneous patient-derived cell sample and define functional overlap between cells from divergent lineages (e.g., PBMCs and neurons). In this respect, computational approaches (e.g., SPADE^53^, viSNE^54^, or CITRUS^55^) which provide high-dimensional representations of deep lineage phenotyping combined with multiple functional measurements represent a valuable means for extracting disease-associated cellular phenotypes from high-content data without relying on prior knowledge. This has been applied to identify cellular phenotypes relevant to prognosis in other disease indications including acute myeloid leukemia (AML)^56^. Such an approach has particular potential, although as yet unapplied, for neuropsychiatric disorders as it is unclear which cell subtypes represent the best functional surrogates for different aspects of CNS pathology or drug discovery indications.

An essential feature of the high-content functional screening approach is the ability to tailor the ligands and cellular readouts used for high-content exploration of patient samples to increase the likelihood of relevant drug target identification. Collectively, G-protein-coupled receptors (GPCRs), ion channels and protein kinases and phosphatases represent the targets for the vast majority of currently approved medications^57^, especially for neuropsychiatric disorders, consistent with their roles as key cellular functional executioners. Thus, targeting these proteins in the drug target discovery phase represents a heuristic means for screening the most ‘druggable’ part of the genome. Importantly, while many of these highly functional cellular proteins, for example GPCRs, are not easily detectable by traditional proteomic screens, an amplified signaling event downstream of these low abundance proteins can be accurately measured using fluorescence flow cytometry or mass cytometry^34^. Furthermore, technologies such as cellular barcoding^58^, which permit multiplexing of the ligand treatments, can be employed to increase the number of functional conditions analyzed in a limited clinical sample, for example 64 concurrent ligand conditions applied to schizophrenia PBMCs^41^. Finally, at the drug discovery stage, candidate compounds can be screened to identify multi-target efficacies, a feature common to existing neuropsychiatric drugs, or potentially toxic off-target interactions directly in patient samples at early stages in the drug development pipeline. The importance of characterizing neuropsychiatric drug interactions outside of conventional targets is poignantly illustrated by the association of TREK-2 potassium channel binding with antidepressant efficacy^59^ or histamine H_1_ receptor affinity with the side-effects of antipsychotic-induced weight gain and sedation^9^. High-content resolution of cellular responses can also be used to explore synergistic interactions between highly specific ligands acting at different sites in the cellular network, a strategy which has shown the potential for overcoming treatment resistance related to genetic heterogeneity in other disease indications such as oncology^60^.

### Drug target prioritization and lead compound validation

One of the major challenges, having identified relevant functional endophenotypes in neuropsychiatric patient samples, is the prioritization of pathway responses with potentially causal disease influences for subsequent drug screening. In this respect, a multi-tiered approach may be useful. First, given the possibility of multiple hits arising from high-content screening (described below) it is important to statistically adjust for false discoveries and extensively cross-validate the findings using techniques which take into account the structure of the data, such as non-parametric permutation procedures and nested cross-validation, as well as to consider primarily functional nodes with exceptional significance in drug-naïve patient vs. control comparisons. Second, target nodes for which activity is correlated to disease severity at baseline (before treatment) or to improvements in symptomatology over the course of efficacious treatment, if longitudinal follow-up samples are available, are more likely to be related to active psychopathology. Third, if genotyping data is available for the same samples, the nodes which correlate to polygenic risk scores, summarizing known genetic risk, or individual risk variants might be suggestive of targets which are supported by parallel genetic evidence, at least in subgroups of patients, and could offer mechanistic insights underlying the endophenotype. Fourth, expression of the target node in brain tissue and/or recapitulation of the target response in brain cell lineages, although not essential, can serve to prioritize targets with CNS activity. This can be further supported by evidence of behavioral abnormalities in animal models, in which the target node has been knocked-out or knocked-in, or developmental changes in transgenic model organisms such as zebrafish^61^. While correlation does not necessarily imply causation, these criteria can serve to prioritize nodes which are more likely to represent causative variants and thus, potentially relevant therapeutic targets. As a final consideration, nodes of comparable significance across these criteria may be chosen based on their amenability to high-throughput drug screening. For example, this may include nodes with a higher signal to noise ratio (Z-prime test), expression in cell-types which are more easily scaled-up in a cost-effective manner and more specific readouts (e.g., protein-epitope phosphorylation) relative to generalized responses (e.g., inflammatory cell proliferation).

A recent study using this approach for drug target discovery in schizophrenia, assessed 3696 cell signaling responses in PBMCs from individuals with schizophrenia and matched controls with a six-week longitudinal follow up^41^. This study prioritized an abnormal response to thapsigargin at PLC-γ1 as the most relevant drug target based on being the most significant node in the drug-naïve patient vs. control comparison, normalization over the course of efficacious clinical antipsychotic therapy, correlation to schizophrenia risk allele loading at the sarcoplasmic/endoplasmic reticulum calcium ATPase 2 (*ATP2A2*) risk locus^10,62^, concurrent activity in neuronal SH-SY5Y cells and parallel evidence of schizophrenia-like behavioral changes in animal models following forebrain-specific ablation of PLC-γ1^63^.

Having prioritized the relevant drug targets from patient-derived cellular models, phenotypic drug screening can be used to identify compounds which normalize these pathway responses and could serve as potential novel drug candidates. This provides a means to identify novel drug candidates even before the full spectrum and functional interactions of putative risk alleles and environmental stressors are defined. For example, one study focused on Timothy syndrome^64^, a disorder caused by a missense mutation in L-type CaV 1.2 calcium channels and associated with developmental delay and autism spectrum condition, showed abnormalities in action potential firing and calcium signaling using patch clamp recording and calcium imaging in iPSC-derived neurons from patients relative to controls. This was further characterized to show differences in calcium-dependent gene expression following depolarization, including tyrosine hydroxylase, with concurrent increases in dopamine and noradrenaline secretion. The authors then screened different L-type calcium channel blockers to show that the tyrosine hydroxylase endophenotype could be improved using roscovitine, a cyclin-dependent kinase inhibitor and atypical L-type channel blocker. Interestingly, in the aforementioned study relating to functional endophenotypes in schizophrenia PBMCs, screening of an FDA-approved compound library (*n* = 786) identified different subsets of L-type calcium channel blockers (e.g., nicardipine, nisoldipine and nimodipine) capable of reversing calcium signaling deficits in response to thapsigargin at PLC-γ1^41^. This highlights this compound class as potentially worthy of follow up across different neuropsychiatric indications, a feature supported by the genetic association of L-type calcium channel subunits (e.g., *CACNA1C* and *CACNB2*) across several major neuropsychiatric disorders^65^.

While this strategy represents a means to rapidly generate early stage candidates, several subsequent steps are relevant when translating these findings towards potential clinical trials. First, novel drug candidates can be directly compared within the same cellular model to established treatments, or to each other, to identify lead compounds which show putative enhanced target specificity, cellular potency or brain penetrance. For example, this has been demonstrated for subtypes of 1,4-dihydropyridines within the L-type calcium channel blocker class in phenotypic screening of functional cellular endophenotypes in schizophrenia^41^. Second, functional endophenotype strategies to date have been modest in terms of sample numbers (discussed below) and validation in larger patient cohorts is necessary to determine whether the target response and drug candidates are reproducible and whether there might be heterogeneity in terms of drug response in the target population. Third, given the overlap in genetic risk factors between different neuropsychiatric disorders, it is important to determine target specificity by comparing target activity in different neuropsychiatric disorders. Previous studies have shown that subsets of abnormalities in cell signaling responses can be shared between different neuropsychiatric disorders while others are unique^51^. Furthermore, this heterogeneity manifests at the individual level whereby individuals with different diagnoses can have partially overlapping signaling profiles. Given the changing diagnostic landscape of neuropsychiatric disorders, it is plausible that targets related to symptom subtypes which extend across diagnostic boundaries could find utility in multiple indications. For example, one study reported that alterations in phosphorylation responses at proinflammatory proteins NF-κB p65 (pS529) and Stat3 (pS727) were shared between conditions with negative symptomatology (schizophrenia and major depression) while aberrant responses to phosphatase inhibitor calyculin A at S6 (pS235/pS236) were shared between conditions with potential psychotic symptomatology (schizophrenia and bipolar disorder)^51^. Conversely, disorders which do not share the same targets can represent relevant exclusion criteria for future clinical trials. Fourth, novel compounds still need to undergo preclinical trials to determine efficacy, toxicity and pharmacokinetics. Despite the limitations of current preclinical models in terms of equating behavioral changes to complex psychiatric symptoms and the reliance on existing treatments as gold standards, functional endophenotypes at least offer the alternative to genetically engineer the target response instead of using acute pharmacological interventions to precipitate symptom-like behaviors. An alternative to the reliance on animal models is the screening of approved medications (drug repurposing) whereby the well documented toxicology, pharmacokinetic, dosing and medicinal chemistry profiles of these compounds could serve to expedite their clinical application to neuropsychiatric indications at a lower cost relative to new chemical entities^66,67^. Finally, in terms of clinical trial design, the same functional endophenotypes used for drug discovery have the potential to serve as ex vivo treatment response predictors, which could stratify patients during clinical drug development to overcome the heterogeneous results of previous clinical trials. Examples include ex-vivo calcium responses at PLC-γ1 in T cells^41^, glucocorticoid sensitivity in whole blood^68^, or CRMP2 phosphorylation in iPSC-derived neurons^52^ correlated with in vivo clinical efficacy in schizophrenia, major depression and bipolar disorder, respectively. In this regard, an increase in the proportion of clinical trials which focus on drug-naïve or recent-onset patients relative to chronic treatment-resistant patients would help to improve the development of effective early intervention strategies. Moreover, where the functional target is sensitive to clinically approved drugs ex vivo, response prediction can be used to validate the target and support the potential in vivo efficacy of novel drugs^41^.

### Limitations and perspective

The functional cellular endophenotype strategy in patient-derived cellular models represents a reverse engineering approach. Traditional target-based, or ‘rational’, drug discovery aims to quantify pathologically-linked gene products and propose a mechanistic drug target using in silico pathway analysis, followed by screening for new drugs in a purpose-built reporter system (e.g., transfected cell line) and inferring clinical relevance. In contrast, the functional endophenotype strategy, proposed here, aims to identify compounds with differential activity directly in physiologically relevant patient-derived cells, relative to healthy individuals, and subsequently dissect their mechanisms of action and underlying genetic targets. Despite the progress made, there are several limitations and key features worth considering to optimize its future utility.

First, obtaining large sample numbers of clinically well-characterized neuropsychiatric patients and sufficient volumes of viable patient-derived cells is a major challenge logistically and in terms of cost. Functional endophenotype studies to date have used relatively few samples, generally less than ten samples for iPSC-based studies^45,69,70^ and up to several dozen samples using PBMCs^41,51,68^, suggesting that they are likely underpowered relative to the complexity of neuropsychiatric phenotypes. The power requirements for target definition using this approach therefore remain to be accurately determined. However, the fact that relatively small endophenotype strategies in schizophrenia PBMCs (*n* = 12 patients for discovery, *n* = 30 patients for validation)^41^ have identified similar lead compounds (L-type calcium channel blockers) as suggested by much larger GWAS studies (*n* = 36,989 patients)^10,66^ raises the possibility that they might have lower power requirements as a result of summarizing genetic risk at the pathway level, a feature echoed by studies using patient iPSCs and cerebral organoids^28,69^. Nevertheless, the increased cost of functional studies on live cells and the possibility of expectancy bias, means that it is important to cross-reference cellular responses with large-scale genetic and proteomic studies such that emerging functional targets might be interpreted in light of better-powered existing studies as the field develops. The effect of cost in limiting sample size is particularly relevant for iPSC-based and organoid studies, where extended culture protocols are needed to reprogram and differentiate cells towards neuronal lineages. In these studies the trade-off between increasing the total number of donors and increasing the number of independent iPSC clones per donor is critical to determining statistical power^69^. Although independent iPSC clones from the same donor are vital to quantifying intra-patient variability (derived from the transformation and differentiation processes), it has been suggested that the use of single iPSC lines for each donor, while maximizing the number of donors, may be the most efficient strategy to maximize statistical power in light of false discovery constraints^69^. Moreover, it is recognized that decreasing inter-patient heterogeneity by focusing on more genetically homogenous patient and control groups might further improve statistical power. This can take the form of selecting patients with highly-penetrant rare genetic variants with a large effect size, patients with high polygenic risk scores based on common variants or gene-editing (e.g., CRISPR-Cas9) to introduce specific risk alleles in isogenic iPSC lines^69,71^. A final consideration in terms of cost is that while cellular assays may initially be more expensive than genotyping or steady-state protein profiling, the resulting functional endophenotypes are more directly amenable to drug screening. In contrast, the interpretation and engineering of genomic or proteomic targets into cellular systems can represent a considerable additional cost beyond the initial target identification.

While sample numbers remain low for patient-derived cellular models in neuropsychiatry, initiatives like the NextGen Genetic Association Studies Consortium, which integrated data from over 2000 iPSC lines with GWAS and QTL data to identify functional cellular phenotypes for cardiovascular disease^72^, suggest that the same upscaling may be possible in the field of mental health. In this respect, composite workflows starting with more accessible cell types (e.g., PBMCs) followed by more resource-intensive cellular systems (e.g., brain organoids), or vice versa, may prove efficient and cost effective. This will likely be complemented by recent efforts to scale-up iPSC-derived cell types for high-throughput compound-screening^22^, inclusion of a greater number of iPSCs from complex idiopathic vs. monogenic disorders and direct comparisons of target overlap between different cellular models from the same individuals. Greater numbers of valuable drug-naïve samples might also be facilitated by including high-risk individuals (e.g., with family history of neuropsychiatric disease) or patient groups where the disease often remains undiagnosed (e.g., major depressive disorder in the context of chronic stress). In line with increasing the power of functional endophenotype strategies, it will also be crucial to leverage available data to control for false discoveries and expectancy bias using statistical methods such as non-parametric permutation procedures and nested cross-validation, which take into account the data structure.

Second, comparing cohorts with high and low polygenic risk profile scores, or with and without rare penetrant risk variants, across key environmental risk factors is an essential step in understanding disease heterogeneity and targeting treatments to specific disease aetiologies. Third, as the diagnostic framework of neuropsychiatric disorders evolves beyond DSM-5 and ICD-10, it will be important to incorporate cellular responses, in addition to other biomarker strategies, to help predict response to clinical treatment on an individual basis and define diagnostic categories which align more closely with therapeutic indications. Fourth, while cellular responses to existing neuropsychiatric treatments can be helpful to validate functional endophenotypes, establish relevant drug discovery workflows and provide clinical correlates for predicted efficacy, the field must eventually depart from the reliance on existing medications in order to identify mechanistically novel drugs which target resistant symptom spectra and avoid the ‘catch-22 scenario’ which has limited the scope of animal models to date.

Finally, disease mechanisms underlying functional cellular endophenotypes require further dissection. Complementary screening technologies such as siRNA, CRISPR-Cas9 genome editing, or protein-specific inhibitors provide opportunities to systematically knock-out or knock-in the function of network proteins to gauge their influence on the target response, as demonstrated in DISC1 iPSC-neuronal models of schizophrenia^73^ or GSK-3β animal models of bipolar disorder^74^. Fluorescence-activated cell sorting of cells from the same patient and cell subtype which differentially exhibit the putative pathological response can also enable characterization of genomic or proteomic readouts whilst controlling for molecular variation between sample donors and cell lineages. Lastly, the combination of technologies such a single-cell RNA sequencing with multiplexed ion beam imaging^75^ in patient-derived brain organoids could provide spatial resolution for understanding the functional interactions between cells which drive neuropsychiatric disease in a physiologically relevant context.

## Conclusion

In conclusion, the presented approach is not the sole solution for addressing the paucity of novel therapeutic options for neuropsychiatric disorders. Its wider applicability, including the pharmacokinetic, brain penetrance and safety profiles of the candidate compounds, remains to be determined in addition to better understanding which neuropsychiatric conditions are likely to best be served by this approach. However, in a field where primary disease tissue is scarcely accessible and genetic complexity is daunting, relative to the magnitude of the public health burden, this approach could offer a complementary strategy to expedite the identification of relevant drug candidates and personalized treatment response predictors.

## Glossary

Cellular coping: The ability of a cell to regulate the effects of a cellular insult or stressor using homeostatic mechanisms.
Drug repurposing: The identification of novel therapeutic indications for drugs which are already approved by regulatory agencies for the treatment of other diseases/disorders.
Functional cellular endophenotype: Abnormal cellular response to a functional ligand in a specific cell subtype, which is shared by subgroups of patients relative to controls, and serves to summarize the effect of complex genetic or environmental risk.
Gene-environment interaction: A different effect of a genotype on disease risk in persons with different environmental exposures.
Genome-wide association study (GWAS): Study which examines the association between a set of genetic variants, distributed across the genome (usually single-nucleotide polymorphisms), and the manifestation of different behavioral or biological traits across individuals in a population.
High-content screening: Method used in biological research and drug discovery to identify substances such as small molecules, peptides or RNAi that alter the phenotype of a cell across multiple parameters.
Induced pluripotent stem cells (iPSCs): Type of pluripotent stem cell that can be generated by reprogramming of adult cells.
Peripheral blood mononuclear cell (PBMC): Type of circulating blood cell with a round nucleus, including lymphocytes (T cells, B cells, NK cells) and monocytes.
